# Pheochromocytoma and heart rate variability: a systematic review and meta-analysis

**DOI:** 10.1016/j.ijcrp.2025.200493

**Published:** 2025-08-19

**Authors:** Frédéric Dutheil, Naira El Gritli, Valentin Magnon, Marek Zak, Reza Bagheri, Julien Steven Baker, Ukadike Chris Ugbolue, Jean-Baptiste Bouillon-Minois, Igor Tauveron, Luc Vialatte

**Affiliations:** a Université Clermont Auvergne, CNRS, LaPSCo, CHU Clermont-Ferrand, Clermont-Ferrand, France; b Université Clermont Auvergne, CNRS, LaPSCo, Physiological and Psychosocial Stress, Clermont-Ferrand, France; c Jan Kochanowski University of Kielce, Institute of Health Sciences, Collegium Medicum, Kielce, Poland; d University of Isfahan, Department of Exercise Physiology, Isfahan, Iran; e Hong Kong Baptist University, Centre for Health and Exercise Science Research, Department of Sport, Physical Education and Health, Hong Kong; f University of the West of Scotland, School of Health and Life Sciences, Institute for Clinical Exercise & Health Science, Glasgow, UK; g Université Clermont Auvergne, iGReD, CNRS, INSERM, CHU Clermont-Ferrand, Endocrinology Diabetology and Metabolic Diseases, Clermont-Ferrand, France; h CHU Clermont-Ferrand, Université Clermont Auvergne, AIST-La prévention active, Clermont-Ferrand, France

**Keywords:** Autonomic nervous system, Biomarkers, Endocrinology, Statistics

## Abstract

**Introduction:**

Pheochromocytoma is a catecholamine-producing tumor, that may alter cardiovascular activity. Conveniently, sympathovagal activity can be measured non-intrusively and pain-free through heart rate variability (HRV).

**Objective:**

To conduct a systematic review and meta-analysis on the impact of pheochromocytoma on HRV parameters.

**Methods:**

PubMed, Cochrane, Embase and Google Scholar were searched until October 10, 2024 for articles reporting HRV parameters in pheochromocytoma patients. Random-effects meta-analysis were conducted on each HRV parameters stratified on pheochromocytoma patients and controls and then comparing these two groups: RR intervals (or Normal-to-Normal intervals-NN), SDNN (Standard Deviation of RR intervals), RMSSD (square root of the mean difference of successive RR intervals), pNN50 (percentage of RR intervals with >50 ms of variation), LF (low-frequency) and HF (high-frequency), and LF/HF.

**Results:**

We included six studies for a total of 178 patients: 94 with pheochromocytoma and 84 controls. Compared to controls, pheochromocytoma patients had higher vagal activity with higher HF (ES = 0.50, 95CI 0.04 to 0.96) and rMSSD (1.22, 0.09 to 2.35), and a tendency for higher pNN50 (1.14, −0.14 to 2.41). The sympathovagal balance tended to be higher in pheochromocytoma patients compared to controls with a tendency for a decreased LF/HF (−0.97, −2.03 to 0.09). Pheochromocytoma patients also tended to have lower RR-intervals than controls (−0.39, −0.86 to 0.07). Sympathetic activity (LF and SDNN) did not differ between pheochromocytoma and controls.

**Conclusion:**

Paradoxically, pheochromocytoma patients have higher HRV. The sympathovagal balance may be explained by a desensitization of beta-adrenergic receptors consecutive to chronic high levels of catecholamine.

## Highlights

• Based on HRV measurements, specific trends are identified between patients with pheochromocytoma and control groups.
• Higher HRV in pheochromocytoma may be explained by catecholamine hypersecretion and autonomic receptor desensitization.
• The study of HRV in the assessment of pheochromocytomas could help monitor cardiovascular health and autonomic function.

## 1 Introduction

Pheochromocytomas are adrenal medulla catecholamine producing tumors arising from chromaffin cells responsible for consequent hemodynamic perturbations especially on cardiovascular function [[1], [2], [3], [4]]. Excessive catecholamine secretion may cause hypertension, orthostatic hypotension, arrhythmia, myocardial hypertrophy, ischemia, cardiomyopathy and in some cases lead to heart failure [[5], [6], [7], [8]]. The panel of these multiple hemodynamic responses have been attributed to cardiac sympathovagal imbalance as well as alpha- and beta-adrenergic receptors and the renin angiotensin aldosterone systems [5]. Indices of heart rate variability (HRV) are reliable indicators of autonomic nervous system modulation. HRV is the fluctuation in the time intervals between two consecutive heartbeats. It reflects regulation of autonomic balance, sympathetic and parasympathetic [9]. A healthy heart is not a metronome. It adapts to changing environment. Indeed, the variability of non-linear systems constantly provides a functionally optimal response to constraints. High frequency (HF) components of HRV are strongly linked to parasympathetic activity whereas low frequency (LF) components are mainly indicative of sympathetic nervous activity [[10], [11], [12], [13]]. The impact of pheochromocytoma on HRV is not clearly understood, and the synthesis of literature has never been done. The role of catecholamine secretion on HRV has been mentioned [[14], [15], [16]]. Furthermore, although sociodemographic and clinical variables such as age, gender, or blood pressure have been associated with HRV parameters in general population [17,18],these associations have not been specifically studied in patients with pheochromocytoma.

Therefore, the aim of this study was to conduct a systematic review and meta-analysis on the impact of pheochromocytoma disease on HRV parameters. A secondary objective was to identify the most explanatory variables.

## 2 Methods

### 2.1 Literature search

**Fig. 1:**
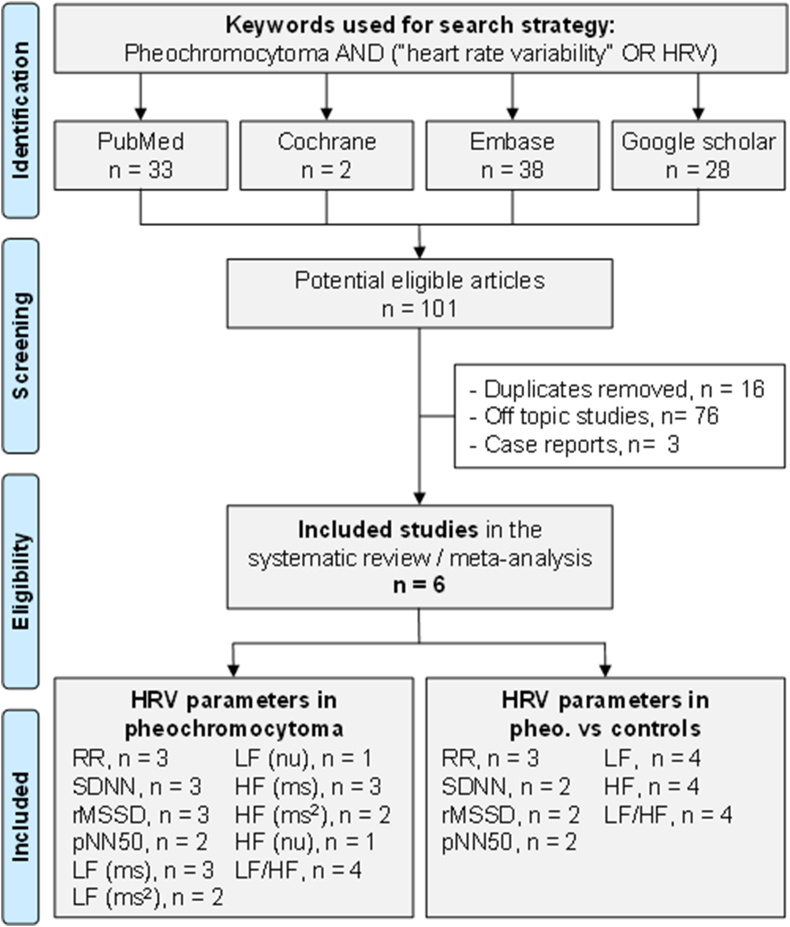
Flow chart.

We reviewed all studies measuring HRV in pheochromocytoma patients. This systematic review was conducted in accordance with the PRISMA (Preferred Reporting Items for Systematic Reviews and Meta-Analyses) recommendations (Appendix 1 and 2). PubMed, Cochrane Library, Embase and Google scholar databases were searched until October 10, 2024, with the following keywords: pheochromocytoma AND (“heart rate variability” OR “HRV”). To be included, studies had to describe our main primary outcome which is the measurement of HRV parameters in pheochromocytoma patients. The exclusion criteria were the following: case reports, duplicates, and studies without frequency or time HRV parameters. Controls without pheochromocytoma were included when available. All articles compatible with our inclusions criteria were included, independently of article language, years of publication, or nature of the control group. Reference lists from all publications meeting the inclusion criteria were manually searched to identify any further studies not found through the electronic search. Ancestry searches were also completed on previous reviews to locate other potentially eligible primary studies. The search strategy is presented in Fig. 1 and in Appendix 3. Two authors (El Gritli Naira and Reza Bagheri) independently conducted the literature searches, collated and reviewed the articles, and extracted the data. When consensus on suitability was not reached, a third author (Frederic Dutheil) reviewed the debated articles. Then, all authors reviewed the eligible articles.

### 2.2 Data extraction

The primary outcome was the analysis of HRV parameters in pheochromocytoma patients. In the time domain, we analyzed RR intervals (or normal-to-normal intervals-NNs), standard deviation of NN intervals (SDNN), percentage of adjacent NN intervals differing by more than 50 ms (pNN50) and the square root of the mean squared difference of successive RR-intervals (RMSSD). The time domain of HRV can be decomposed into its frequency components by the spectral analysis technique, either with the fast Fourier transform algorithm or with autoregressive modeling. This is analogous to a prism that refracts light into its wavelength components. In the spectral domain, we analyzed low frequency (LF, 0.04 ± 0.15 Hz), high frequency (HF, 0.15 ± 0.4 Hz), and the LF/HF ratio. LF and HF powers are absolute powers, reported in units of ms^2^ (square milliseconds). LF nu and HF nu are normalized powers, called relative powers, in the LF and HF bands, a derived index that is calculated by dividing LF or HF by an appropriate denominator representing the relevant total power: LF nu = LF/(LF + HF) and HF nu = HF/(LF + HF). The normalized powers enable direct comparison of the frequency domain measurements of two patients despite a large variation in specific band power and total power. LF power represents both sympathetic and parasympathetic activity and is associated with SDNN, but LF nu emphasizes the control and balance of cardiac sympathetic behavior [19]. HF power and HF nu represent the most efferent parasympathetic activity and are associated with RMSSD and pNN50 [20]. Such as for SDNN, both sympathetic and parasympathetic activities contribute to very low frequency (VLF) with uncertainty about the physiological mechanisms responsible for activity in this band [21]. The LF/HF ratio is the most sensitive indicator of sympathovagal balance [19] which was also calculated and reported in this meta-analysis. A lower LF/HF ratio indicates a higher sympathovagal balance. Secondary outcomes included pheochromocytoma characteristics (plasma and urinary catecholamines) and clinical parameters (age, gender, heart rate, blood pressure).

### 2.3 Quality of assessment

**Fig. 2:**
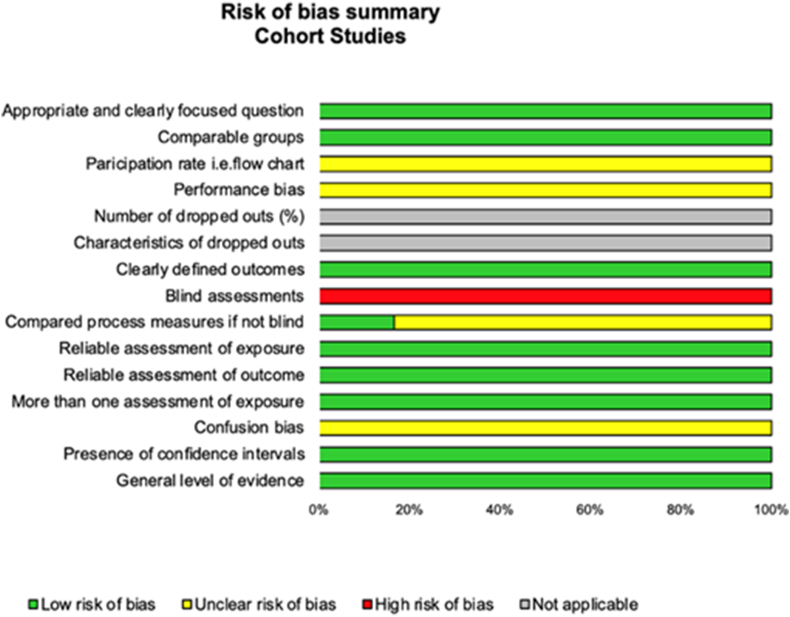
Methodological quality of included studies using the SIGN checklist.

We used the Scottish Intercollegiate Guidelines Network (SIGN) criteria to check the quality of included articles with the dedicated evaluation grids. The SIGN score for cohort and cross-sectional studies has two sections: internal validity (14 items) and overall assessment of the study (3 items), with 4 possibilities of answers per item (yes, no, can't say or not applicable) (Fig. 2 and Appendix 4) [22]. We also used “STrengthening the Reporting of OBservational studies in Epidemiology” (STROBE) for checking the reporting quality of cohort's studies [23]. The STROBE statement is a checklist of 22 items related to the title/abstract, introduction, methods, results and discussion sections of articles. Six of the 22 items are spitted into several sub-items. We attributed one point per item or sub-item, to achieve a maximal score of 32 and then calculated [23].

### 2.4 Statistical considerations

We conducted meta-analyses on the effect of pheochromocytoma on HRV parameters. For the statistical analysis, we used Stata software (version 16, StataCorp, College Station, US) [[24], [25], [26], [27]]. Main characteristics were synthetized for each study population and reported as the mean ± standard–deviation (SD) for continuous variables and the number (%) for categorical variables. We conducted random effects meta–analyses (DerSimonian and Laird approach) [28] on each HRV parameter (RR-intervals, SDNN, rMSSD, pNN50, LF, HF, LF/HF), stratified on pheochromocytoma patients and controls – results being expressed in the unit of each HRV parameter. Then, we conducted meta-analyses on each HRV parameter in pheochromocytoma patients compared to controls – results being expressed in effect size (ES, standardised mean differences – SMD) [29]. An ES is a unitless measure, centred at zero if the HRV parameter did not differ between pheochromocytoma patients and controls. A negative effect size (ES, standardised mean differences - SMD) denoted lower HRV in pheochromocytoma patients than in controls. An ES of −0.8 reflects a large effect i.e. a large HRV decrease in patients compared to controls, −0.5 a moderate effect, and −0.2 a small effect. For parameters that could be expressed in different units such as LF and HF, we stratified results by unit (ms, ms2, nu). When a study reported several units for the same parameter, only one unit was chosen to preserve the weighting of studies in the overall results for each parameter [30,31]. We evaluated heterogeneity in the study results by examining forest plots, confidence intervals (CI) and I-squared (I^2^). I^2^ measures heterogeneity of results between studies, and ranges from 0 to 100 %. I2<25 % reflects a low heterogeneity, 25<I2<50 % a modest and I2>50 % a high heterogeneity. A high heterogeneity between studies may be due to variations in study populations, different inclusion/exclusion criteria or different methods of HRV assessment. We verified the strength of our results by conducting further meta-analyses (sensitivity analyses) after exclusion of studies that were not evenly distributed around the base of the meta-funnels, as well as depending on HRV measurement protocols (e.g., 24-h Holter vs. resting ECG). When possible (sufficient sample size i.e. more than 5 studies), meta regressions were proposed to study the relation between changes in HRV parameters (RR intervals, RMSSD, pNN50, SDNN, LF, HF, LF/HF) and clinically relevant parameters such as gender, age, systolic blood pressure, diastolic blood pressure and blood and urinary catecholamines. Results were expressed as regression coefficients and 95CI. P values less than 0.05 were considered statistically significant.

## 3 Results

An initial search produced 101 potential eligible articles (Fig. 1). Removal of duplicates and use of the selection criteria reduced the number of articles reporting the evaluation of HRV in pheochromocytoma patients to six articles [[14], [15], [16],[32], [33], [34]]. Both reviewers independently retrieved the same articles (100 % agreement, kappa = 1). All included articles were written in English. Main characteristics of the studies are resumed in Table 1.

**Table 1:** Characteristics of included studies.

Study	Objectives	Country	Design	Pheochromocytoma patients				Controls				ECG (min)	HRV parameters	HRV measures	Other parameters
				n	Age, years	Sex, %men	BP, mmHg	n	Age, years	Sex, %men	BP, mmHg				
Dabrowska, 1995a	To evaluate HRV in patients with pheochromocytoma	Poland	Cohort	13	42 ± 14.4	15	138/85 ± 25/14	13	42 ± 14.4	15	133/85 ± 18/11	1440	RR, SDNN, rMSSD, pNN50, LF, HF, LF/HF	Before surgery	Urinary catecholamines/24h
Dabrowska, 1995b	1)To evaluate the sympathovagal balance before and during sudden BP elevations or before episodes of complex cardiac arrhythmias in patients with pheochromocytoma, and 2) to compare the results with matched patients with essential hypertension	Poland	Cohort	4	40.8 ± 9.2^a^	10	138/86 ± 17/9^a^	3	40.4 ± 6.9^a^	10^a^	131/85.6 ± 11/12^a^	10	RR, SDNN, rMSSD, pNN50, LF, HF, LF/HF	1 h before blood pressure elevations	Urinary catecholamines/24h
				4	40.8 ± 9.2^a^	10	138/86 ± 17/9^a^	3	40.4 ± 6.9^a^	10^a^	131/85.6 ± 11/12^a^	5		During blood pressure elevations	
				4	40.8 ± 9.2^a^	10	138/86 ± 17/9^a^	–	–	–	–	10		1 h before arrhythmias	
Dabrowska, 1995c	To assess the effect of alpha-adrenergic blockade in patients with pheochromocytoma on the heart rate and its variability	Poland	Observa-tional	17	43.4 ± 54^a^	23^a^	138/85 ± 24/15	–	–	–	–	20	RR, SDNN, rMSSD, pNN50, LF, HF, LF/HF	Before alpha- adrenergic blockade	Urinary catecholamines/24h, QT, ventricular arrhythmias
				17	43.4 ± 54^a^	23^a^	127/79 ± 16/12	–	–	–	–	20		During alpha- adrenergic blockade	
Moriguchi, 1993	To evaluate the pathogenesis of orthostatic hypotension using HRV	Japan	Cohort	3	62 ± 3.5^a^	57	137/79 ± 7/3^a^	15	63 ± 7.7	60	138/85 ± 12/12	10	LF, HF, LF/HF	At rest in the supine position	Plasma catecholamines
Munakata, 1999	To compare BP and HRV response to orthostatic stress between patients with pheochromocytoma to controls (normotensive and essential hypertension patients)	Japan	Cross sectional	12	36 ± 10.4	50	142/78 ± 35/7	30	34 ± 2	37	109/52 ± 11/11	10	RR	At rest in the supine position	Respiratory power
Sesay, 2008	1) To assess the sympathovagal activity using real-time HRV, and 2) the relation between HRV and catecholamine release during laparoscopic adrenalectomy for pheochromocytoma	France	Cohort	20	52.7 ± 15.9	50	120/- ±25/-	20	53.6 ± 14.5	50	110/- ±30/-	30	LF, HF, LF/HF	Before and after induction of anesthesia	Plasma catecholamines

a extrapolated data.

### 3.1 Study designs of included studies

Among the six studies included, one was cross-sectional [34], four were cohort prospective [[14], [15], [16],^32^] and one was observational [33]. Included studies were published from 1993 to 2008 and conducted across three countries (Poland [16,32,33], Japan [15] and France [14]).

### 3.2 Quality of articles

Using SIGN, mean quality score of the included studies was 60.0 ± 2.6 % for Yes responses, varying from 58.8 % [14,16,33,34] to 64.7 % [32] (Fig. 2 and Appendix 4). No studies had blind assessments. The quality score also decreased due to confusion bias, and lack of details about participants rate and selection process (absence of flow chart) [14,16,34]. Using STROBE, scores were 65.2 ± 12.8 % ranging from 48.8 % [16] to 77.5 % [33] (Appendix 4).

### 3.3 Aims of included articles

All included articles aimed to compare HRV between patients with pheochromocytoma and controls [[14], [15], [16],^32^,^34^] except one study that aimed to assess the effect of alpha-adrenergic blockade in patients with pheochromocytoma on the heart rate and its variability [33]. Two studies were interested in orthostatic hypotension: one using HRV to study the pathogenesis of orthostatic hypotension [15] and one comparing blood pressure and HRV response to orthostatic stress between pheochromocytoma patients and controls [34]. One study evaluated the sympathovagal balance before sudden blood pressure elevations or arrhythmias in pheochromocytoma patients [32]. Finally, one study assessed the relation between sympathovagal activity and catecholamine release during laparoscopic adrenalectomy for pheochromocytoma [14].

### 3.4 Inclusion and exclusion criteria

All studies included adults with pheochromocytoma and controls, except one study without a control group [33]. Pheochromocytoma patients and controls shared the same exclusion criteria, and were paired on age [[14], [15], [16],[32], [33], [34]], gender [[14], [15], [16],[32], [33], [34]], and forms of hypertension (transient or persistent) [16]. Four studies described other exclusion criteria: left ventricular hypertrophy [16], diabetes, heart and neurological disease [14], orthostatic hypotension [15,34], cardiovascular medication two weeks prior the study [15,34], and cigarettes and caffeine beverages two weeks before [34].

### 3.5 Population

Sample size ranged from 3 [15] to 30 [34], for a total of 178 patients: 94 with pheochromocytoma and 84 controls.

Age was described in all studies except one [32]. The mean age was 51.2 years old (95CI 46.2–56.3 years) in pheochromocytoma patients, ranging from 36.0 [34] to 62.0 years [15], and 45.3 years old (39.7–51.0 years) in controls, ranging from 34.0 [34] to 63.0 years [15].

Sex was described in all studies. The proportion of men was 25 % (18–32 %) in pheochromocytoma patients ranging from 8 % [16] to 67 % [15], and 24 % (18–30 %) in the controls, ranging from 5 % [32] to 48 % [15].

Blood pressure was described in all studies. Mean systolic and diastolic blood pressure was 128/79 mmHg (119/74 to 137/83 mmHg) in pheochromocytoma patients, ranging from 101/62 [15] to 170/100 mmHg [16], and 123/75 mmHg (115/66 to 130/84 mmHg) in controls, ranging from 104/52 [34] to 138/87 mmHg [15].

Heart rate was described in three studies [14,15,33]. Mean heart rate was 76 beats per minute (71–81 bpm) in pheochromocytoma patients, ranging from 65 [14] to 89 bpm [33], and 72 bpm (65–80 bpm) in controls, ranging from 65 [14] to 78 bpm [15].

Other characteristics were seldomly reported such as blood [14,15] or urinary [16,32,33] levels of catecholamines, measured with high performance liquid chromatography and fluorometry respectively.

### 3.6 Characteristics of pheochromocytoma patients

Diagnosis of pheochromocytoma was established on clinical (headaches, hypertension, sweating and palpitations), biochemical (catecholamines) and radiological (tomographic scan and 131I-metaiodobenzylguanide scintigraphy) criteria in one study [14]. Diagnosis was proven histologically in four studies [16,[32], [33], [34]] with additional biology (catecholamines) in three studies [16,32,33]. One study did not detail the criteria of diagnosis of pheochromocytoma [15]. Only one study reported a malignant pheochromocytoma in two of their patients [34]. Studies did not indicate the duration of pheochromocytoma patients’ symptoms.

### 3.7 HRV monitoring

HRV was measured using ECG at a resting supine position in three studies [14,15,34] and using 24- hour holter-ECG in three studies [16,32,33]. All studies retrieved HRV over periods of 5–20 min, except one that used only the whole 24-h recording [16]. ECG were monitored using devices [35,36] were Oxford MR-14 or MR-45 2 channel tape recorders (Abingdon, UK) and analyzed by an Oxford Medilog Excel 2 device in three studies [16,32,33]. The rest of them had a distinct Holter monitoring/software system: Datex (Contec TM, Osaka, Japan)/MemCalc/Tawara TM Suwa Trust, Tokyo, Japan [14], CBM-3000, Nippon Colin, Aichi, Japan/personal computer, NECPC-9801RX, Nippon Electronic Co, Tokyo, Japan [15] and personal computer/Carspan computer program [34]. Patients with artefacts beats, episodes of wandering atrial pacemaker and arrhythmias were excluded from the evaluation in all studies.

### 3.8 HRV parameters

HRV was analyzed through both time and frequency domains in most studies, except two studies that only reported frequency domain [14,15] and one study only one variable of time domain [34]. For time domain parameters, RR intervals was determined in four studies [16,32,33], and SDNN, rMSSD and pNN50 in three studies [16,32,33]. For frequency domain parameters, LF, HF and LF/HF were determined in five studies [[14], [15], [16],^32^,^33^]. Moreover, LF and HF components of HRV were determined in three studies using fast Fourier transformation and expressed as a spectral amplitude in milliseconds in preference to ms2 (power) as it does not require a logarithmic transformation [16,32,33]. In one study, the LF and HF were expressed both in absolute units of power (ms2) and normalized units (nu) [14]. No study reported neither total power nor VLF.

### 3.9 Meta–analyses on levels of HRV parameters (absolute values)

**Fig. 3:**
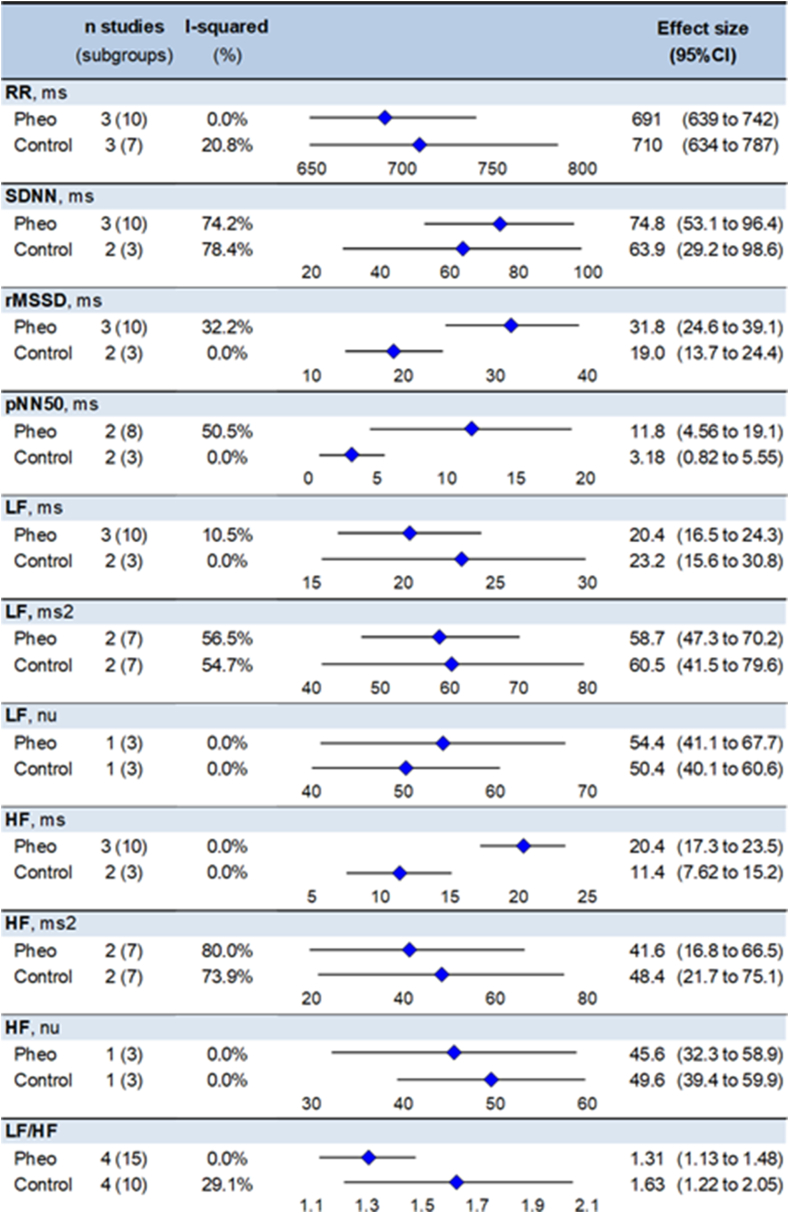
Meta-analysis of HRV parameters of pheochromocytoma patients and controls.

RR-intervals were at 691 ms in pheochromocytoma patients (95CI 639–742 ms) and at 710 ms in controls (634–787 ms), SDNN at 74.8 ms (53.1–96.4 ms) in pheochromocytoma and 63.9 ms (29.2–98.6 ms) in controls, rMSSD at 31.8 (24.6–39.1 ms) and 19 ms (13.7–24.4 ms), pNN50 at 11.8 ms (4.56–19.1 ms) and 3.18 ms (0.82–5.55 ms), LF at 24.4 ms (16.5–24.3 ms) and 23.2 ms (15.6–30.8 ms), LF at 58.7 ms2 (47.3–70.2 ms2) and 60.5 ms2 (41.5–79.6 ms2), LF at 54.4 nu (41.1–67.7 nu) and 50.4 nu (40.1–60.6 nu), HF at 20.4 ms (17.3–23.5 ms) and 11.4 ms (7.62–15.2 ms), HF at 41.6 ms2 (16.8–66.5 ms2) and 11.4 ms2 (7.62–15.2 ms2), HF at 45.6 nu (32.3–58.9 nu) and 49.6 nu (39.4–59.9 nu), LF/HF at 1.31 (1.13–1.48) and 1.63 (1.22–2.05), respectively. SDNN pNN50, and LF and HF in ms2 had a degree of heterogeneity >50 % (Fig. 3 and Appendix 5) [37].

### 3.10 Meta–analyses comparing pheochromocytoma to controls (effect sizes)

**Fig. 4:**
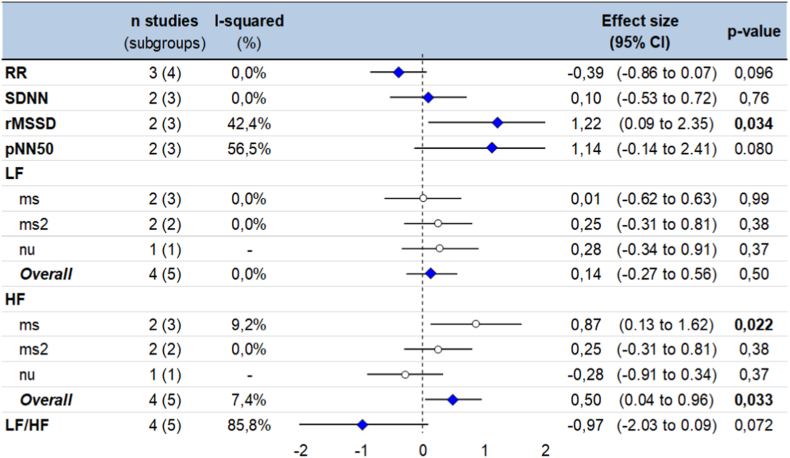
Meta-analysis of HRV parameters of pheochromocytoma patients compared to controls.

Compared to controls, pheochromocytoma patients had higher vagal activity with higher HF (ES = 0.50, 95CI 0.04 to 0.96, p = 0.033) and rMSSD (1.22, 0.09 to 2.35, p = 0.034), and a tendency for higher pNN50 (1.14, −0.14 to 2.41, p = 0.080). The sympathovagal balance tended to be higher in pheochromocytoma patients compared to controls with a tendency for a decreased LF/HF (−0.97, −2.03 to 0.09, p = 0.072). Pheochromocytoma patients also tended to have lower RR-intervals then controls (−0.39, −0.86 to 0.07, p = 0.096). Sympathetic activity (LF and SDNN) did not differ between pheochromocytoma and controls (Fig. 4 and Appendix 6) [37]. Majority of the meta-analyses had a low degree of heterogeneity (I2<10 %), except for pNN50 and LF/HF that had an I2 >50 %).

### 3.11 Meta–regressions and sensitivity analyses

**Fig. 5:**
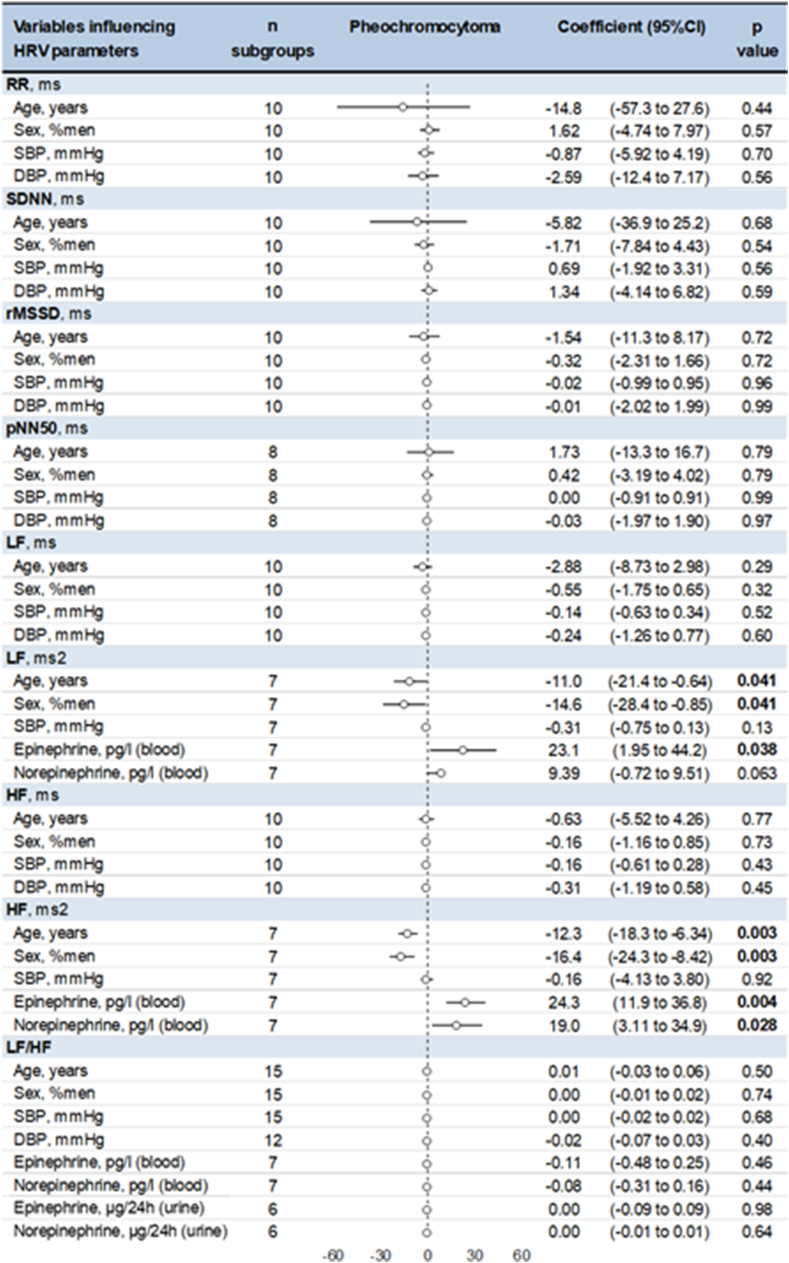
Meta-regressions of significant factors influencing HRV in pheochromocytoma patients.

In pheochromocytoma patients, age and male gender were associated with lower LF ms2 (coefficient −11.0, 95CI −21.4 to −0.64, and −14.6, −28.4 to −0.85, respectively) and lower HF ms2 (12.3, −18.3 to 6.34, and −16.4, −24.3 to −8.42, respectively) without influence on LF/HF. In addition, blood epinephrine was associated with higher LF ms2 (0.02, 0.002 to 0.04) and HF ms2 (0.02, 0.01 to 0.04), and blood norepinephrine was associated with higher HF ms2 (0.02, 0.003 to 0.03) (Fig. 5). Results were similar in controls, with age being associated with lower LF and HF in ms2, male gender being associated with lower LF and HF in ms2 and LF/HF, and blood epinephrine and norepinephrine being associated with higher LF and HF. Systolic blood pressure was also associated with higher HF and LF/HF in controls (Appendix 7) [37]. No other meta regressions were significant for pheochromocytoma patients and for controls, as well as for factors influencing HRV in pheochromocytoma compared to controls (Appendix 8) [37].

Funnel plots of meta-analyses analyzing for potential publication bias are presented in Appendix 9 [37]. Meta–analyses were reperformed after the exclusion of studies that were not evenly distributed around the base of the funnel and showed similar results (data not shown). As only one study used only the whole 24-h-Holter recording [16], we also computed sensitivity analyses using only HRV measures <30 min, which gave similar results (Appendix 10).

## 4 Discussion

This study highlighted a higher parasympathetic activity in patients with pheochromocytoma compared to controls, without difference in terms of sympathetic activity. Moreover, in pheochromocytoma patients, age, sex and blood catecholamines seemed to influence HRV components. Finally, we discussed clinical and therapeutical applications.

### 4.1 The impact of pheochromocytoma on HRV

Impact of pheochromocytoma on HRV parameters remain controversial. Our results are similar to a previous study that showed a higher parasympathetic tone in pheochromocytoma patients compared to those with primary hypertension [16]. This high vagal tone may be caused by prolonged reflex stimulation of the parasympathetic nervous receptors [16]. Another reason may be that rising blood pressure stretches the baroreceptors in the carotid sinuses, leading to afferents via the glossopharyngeal nerves, and potentially triggering inhibition of sympathetic activity as well as activation of the parasympathetic [38]. Moreover, we did not find any significant change in sympathetic activity between pheochromocytoma patients and controls, which may be explained by a desensitization of the sympathetic cardiovascular receptors [39]. In humans and rats suffering from pheochromocytoma, prolonged stimulation of sympathetic tone has been related to a decrease density of alpha- and beta-adrenergic receptors [40,41]. Conversely, several studies showed an increased cardiac sympathetic tone through an excessive alpha-adrenergic stimulation by the tumor [15,32]. Excessive release of catecholamines from the adrenal tumor has indeed been described to be one explanation of the pathogenic sources of hypertension and cardiac abnormalities such as arrhythmias, myocardial hypertrophy ischemia and cardiomyopathy seen in patients with pheochromocytoma [5,39]. However, this observation is not consistent as an increase of plasma catecholamine levels are not correlated systematically to elevated arterial blood pressure [19,41]. Another element of discussion is that some studies suggests that low frequency component reflects both sympathetic and parasympathetic activity and no specific recommendation for its interpretation has been provided [42].

### 4.2 Other variables linked to HRV in pheochromocytoma

Age and male gender were associated with both lower LF ms^2^ and HF ms^2^ without influence on LF/HF in pheochromocytoma patients similarly as it was demonstrated respectively in healthy patients [43], in hypertension patients [44] and in our control population. In addition, we also demonstrated a significant association between catecholamines and HRV frequency domains. Blood epinephrine was associated with higher LF ms^2^ and HF ms^2^ and blood norepinephrine was associated with higher HF ms^2^. Thus, it may suggest that an excessive chronic catecholamine secretion induces a desensitization of beta-adrenergic receptors leading to a better responsiveness of autonomic activity [16]. Finally, surprisingly systolic blood pressure was associated with higher HF and LF/HF in controls and thus an increase in parasympathetic activity with no correlation found in pheochromocytoma patients. In the literature, results were controversial in term of the effect of hypertension on HRV parameters [35].

### 4.3 Clinical and therapeutical applications

HRV is a popular and widely used clinical tool today. Its use has increased significantly in many areas of cardiology [34]. For example, several authors have examined the link between HRV and blood pressure [35,36]. Others use HRV to study cardiovascular risk [37,38]. More generally, many research articles seek to understand the relationship between HRV and human pathologies, to prevent them in advance and detect abnormalities early [39,40]. The fact that it reflects the balance between the branches of the nervous system enables it to play a major role in prevention and makes it a reliable indicator of overall health. HRV has great potential for analyzing fluctuations in the autonomic nervous system. Research into HRV contributes to a better understanding of physiological mechanisms, the effects of drug treatments and pathological processes [19]. Assessment of HRV parameters in pheochromocytoma patients might be clinically relevant. Indeed, patients with low vagal tone are more prone to heart abnormalities such as rhythm disorders, hypertension and myocardial cells damage [45]. Moreover, even though total power, another HRV variable was not available in our study, it has been shown to be predicted to elevated risk cardiac morbimortality [46,47]. Therefore, these data suggests that HRV measurement may be used as a tool in the evaluation of the severity of heart dysfunction and overall health of these patients. In addition, although it is known that an increase in HRV indicates a better adaptation to microenvironment changes [10], one study demonstrated that HRV parameters representing both sympathetic and parasympathetic activities significantly decreased during non-selective alpha-adrenergic blockade in pheochromocytoma patients and showed a decrease in blood pressure elevations and arrhythmias [33]. Thus, this suggests that having a lower HRV may be relevant in certain clinical contexts, as it can suppress ventricular arrhythmias in pheochromocytoma patients.

### 4.4 Limitations

Some limitations should be acknowledged. Our meta-analysis inherits the limitations of all meta-analysis [36,48,49]. Selection bias might affect the analysis as the number of included studies is moderate. However, the use of electronic bibliographic databases with no date or language-based exclusion criteria minimized the risk of missing studies. Moreover, publication bias is limited since the analyses of the funnel plots revealed a homogenous distribution of included studies. In addition, no randomized controlled trial was included in this meta-analysis. Most of the studies were cohort single institutions with a small number of enrolled patients suggesting that the quality of the evidence is not high and thus limiting the generalizability of our results. Furthermore, despite data collection not being reported uniformly (measurement conditions of HRV such as resting ECG or 24-h Holter, units such as for LF and HF, inclusion criteria, health status of controls), although similar, we demonstrated significant difference in the impact of pheochromocytoma on HRV parameters. Finally, characteristics of study participants such as clinical, biochemical, severity or duration of pheochromocytoma, drugs and comorbidities (smoking, diabetes etc) were poorly reported. This is particularly true of the duration of the disease, which is rarely mentioned even though it could influence the desensitization of adrenergic receptors [40]. Similarly, drug treatments were often absent or partially described, as were lifestyle habits (smoking, caffeine) even though they may have a direct impact on HRV [50,51]. As a result, we could not assess several variables that could potentially contribute to explain heterogeneity. However, this is the first meta-analysis on this topic.

## 5 Conclusion

Paradoxically, pheochromocytoma patients have higher HRV. The sympathovagal balance may be explained by a desensitization of beta-adrenergic receptors following chronic high levels of catecholamine. The benefits of HRV assessment in the evaluation and monitoring of the severity of pheochromocytoma should be further investigated, given its potential as a noninvasive, reliable, and pain-free measure.

## CRediT authorship contribution statement

**Frédéric Dutheil:** Writing – original draft, Validation, Supervision, Resources, Project administration, Methodology, Formal analysis, Conceptualization. **Naira El Gritli:** Writing – original draft, Visualization, Software, Methodology, Investigation, Formal analysis. **Valentin Magnon:** Writing – review & editing, Visualization, Software, Methodology, Investigation, Formal analysis, Data curation. **Marek Zak:** Writing – review & editing, Validation, Methodology, Investigation. **Reza Bagheri:** Writing – review & editing, Methodology, Investigation. **Julien Steven Baker:** Writing – review & editing, Validation. **Ukadike Chris Ugbolue:** Writing – review & editing, Methodology, Investigation, Formal analysis. **Jean-Baptiste Bouillon-Minois:** Writing – review & editing, Resources, Methodology, Investigation, Data curation. **Igor Tauveron:** Writing – review & editing, Visualization, Validation, Software, Methodology, Formal analysis, Conceptualization. **Luc Vialatte:** Writing – review & editing, Visualization, Validation, Software, Methodology.

## Disclosure statement

The authors have nothing to disclose.
